# Methylated Flavonols from *Amomum koenigii* J.F.Gmel. and Their Antimicrobial and Antioxidant Activities

**DOI:** 10.1155/2020/4812312

**Published:** 2020-02-18

**Authors:** Minh Giang Phan, Thi Viet Huong Do, Quoc Binh Nguyen

**Affiliations:** ^1^Faculty of Chemistry, VNU University of Science, Vietnam National University, Hanoi, 19 Le Thanh Tong Street, Hanoi, Vietnam; ^2^Vietnam National Museum of Nature, Vietnam Academy of Science and Technology, 18 Hoang Quoc Viet Street, Hanoi, Vietnam

## Abstract

Methylated flavonols form a special group with modulating biological activities in comparison with kaempferol and quercetin. The present study isolated ten compounds including two kaempferol methyl ethers: 5-hydroxy-3,7,4′-trimethoxyflavone (**1**), 3-hydroxy-5,7,4′-trimethoxyflavone (**6**); four quercetin methyl ethers: retusin (5-hydroxy-3,7,3′,4′-tetramethoxyflavone) (**4**), 3,5-dihydroxy-7,3′,4′-trimethoxyflavone (**5**), 3,4′-dihydroxy-5,7,3′-trimethoxyflavone (**7**), and 3,5,7,3′,4′-pentamethoxyflavone (**9**); *β*-sitosterol (**2**); 5-hydroxy-1-(4′-hydroxyphenyl)eicosan-3-one (**3**); *p*-hydroquinone (**8**); and vanillic acid (**10**) from the rhizomes and fruit of *Amomum koenigii* J.F.Gmel. (Zingiberaceae). Their structures were determined by MS, NMR, and X-ray spectroscopic techniques. Among the methylated flavonols, **1**, **4**–**7**, and **9** were isolated for the first time from the rhizomes, while **1**, **4**, and **5** were isolated from the fruit. Compounds **2**, **3**, **7**, **8**, and **10** were reported for the first time from the species. Three main methylated flavonols **1**, **4**, and **5** were quantitatively analyzed in the rhizomes of *A. koenigii* by RP-HPLC-DAD; their contents were determined to be 1.81% (**1**), 1.38% (**4**), and 1.76% (**5**). The antimicrobial assay against *Escherichia coli*, *Pseudomonas aeruginosa*, *Bacillus subtilis*, *Staphylococcus aureus*, *Aspergillus niger*, *Fusarium oxysporum*, *Candida albicans*, and *Saccharomyces cerevisiae* and antioxidant DPPH scavenging test were performed for the isolated methylated flavonols.

## 1. Introduction

*Amomum* Roxb. is a large genus with about 250 species and belongs to Zingiberaceae. In recent years, 21 *Amomum* species have been recorded in Vietnam [1]. The chemistry of a few *Amomum* species from Vietnam has been studied [2–4]. The fruit of *A. koenigii* has been used as an aromatic stomachic in China, India, and Thailand [5, 6]. A phytochemistry report on *A. koenigii* in China describes the isolation of eicosenones and methylated flavonols from the fruit of *A. koenigii* [5]. We investigated the occurrence of methylated flavonols in the fruit and rhizomes of *A. koenigii* from Vietnam (Figures [Supplementary-material supplementary-material-1] and [Supplementary-material supplementary-material-1]) and isolated six methylated flavonols from the rhizomes for the first time. Their structures were analyzed by MS and 1D NMR spectroscopic techniques; the positions of methyl groups were determined by 2D NOESY and X-ray techniques. The contents of the main flavonoids in the rhizomes were analyzed by using RP-HPLC-DAD, and their antimicrobial and antioxidant activities were evaluated.

## 2. Materials and Methods

### 2.1. General Experimental Procedure

ESI-MS spectra were measured on a Thermo Fisher Scientific LTQ Orbitrap XL mass spectrometer in CH_3_OH solution. ^1^H-NMR, ^13^C-NMR, and DEPT spectra were recorded on a Bruker Avance 500 NMR spectrometer at 500 MHz for proton and 125 MHz for carbon-13. Tetramethyl silane (TMS) was used as the NMR internal standard. Diaion HP-20 (Mitsubishi, Japan) and silica gel (Merck, Germany) of 40–63 and 15–40 *μ*m were used for column chromatography (CC). Thin-layer chromatography (TLC) was performed on Merck precoated aluminum silica gel 60 F_254_ plates. Tryptone soya agar (TSB, Sigma-Aldrich, USA) and Sabouraud's dextrose agar (SDB, Sigma-Aldrich, USA) were used for the antimicrobial activity assay. Reagents and standard compounds used in the antioxidant DPPH scavenging assay were purchased from Sigma-Aldrich (USA).

### 2.2. Plant Material

The fresh fruit (3 kg) and rhizomes (16 kg) of *A. koenigii* were collected in July 2016 from Chu Yang Sin National Park, Hoa Son Commune, Krong Bong District, Dak Lak Province, Vietnam (coordinates: from 12°14′16″ north to 13°30′58″ north and from 108°17′47″ west to 108°34′48″ west). The plant material was identified by Dr. Quoc Binh Nguyen, Vietnam National Museum of Nature, Vietnam Academy of Science and Technology, Hanoi, Vietnam. A voucher sample (No. AK-7-17) was deposited in the same museum.

### 2.3. Extraction and Isolation

#### 2.3.1. The Rhizomes

The rhizomes were air-dried and then oven-dried at 40–50°C. The dried material was ground into powder. The powder (2.7 kg) was macerated with MeOH at room temperature three times, each time for 7 days. The extracts were filtered and concentrated under reduced pressure to give the MeOH extract. The MeOH extract was suspended in water, and the water phase was extracted with *n*-hexane and dichloromethane. Evaporation of solvents under reduced pressure gave *n*-hexane- and dichloromethane-soluble fractions. The *n*-hexane- and dichloromethane-soluble fractions were combined based on their TLC similarities, and 20.2 g of the combined fraction was separated by CC over silica gel, eluting with *n*-hexane-acetone 19 : 1, 15 : 1, 9 : 1, 5 : 1, and 1 : 1 (v : v) to give 11 fractions (Fr. 1–Fr. 11). Fr. 2 (0.83 g) was purified by CC over silica gel, eluting with *n*-hexane-EtOAc 49 : 1, 29 : 1, 19 : 1, 9 : 1, and 6 : 1 to give **1** (112 mg) and **2** (20.1 mg). Frs. 4 and 5 were combined (0.77 g) and separated by CC over silica gel, eluting with *n*-hexane-acetone 20 : 1, 15 : 1, 12 : 1, 9 : 1, and 3 : 1 to yield **3** (5 mg). Fr. 7 (1.73 g) was separated by CC over silica gel, eluting with *n*-hexane-acetone 20 : 1, 15 : 1, 9 : 1, 6 : 1, 3 : 1, and 1 : 1 to yield **1** (36.9 mg), **4** (8.2 mg), and **5** (25.4 mg). Frs. 8 and 9 were combined (3.15 g) and separated by CC over RP-18, eluting with 70%, 80%, and 90% MeOH-H_2_O and MeOH. The fraction eluting with MeOH was separated by CC over silica gel, eluting with *n*-hexane-acetone 15 : 1, 12 : 1, 9 : 1, 6 : 1, and 3 : 1 to give **1** (5 mg). Fr. 10 (2.41 g) was separated by CC over RP-18, eluting with 70%, 80%, and 90% MeOH-H_2_O and MeOH. The fraction eluting with 70% MeOH-H_2_O (1.56 g) was separated by repeated CC over silica gel, eluting with *n*-hexane-acetone 25 : 1, 20 : 1, 12 : 1, 9 : 1, 6 : 1, and 3 : 1 to give **4** (2 mg) and **5** (20 mg). Fractions eluting with 80% and 90% MeOH-H_2_O were combined (0.54 g) and separated by CC over silica gel, eluting with *n*-hexane-EtOAc 12 : 1, 9 : 1, 6 : 1, 3 : 1, 1 : 1, and 1 : 2 to give **4** (5 mg), **6** (7.3 mg), **7** (30.2 mg), **8** (3 mg), and **9** (6 mg).

#### 2.3.2. The Fruit

The fruit was washed, air-dried to remove surface water, and then crushed. The crushed fruit (3 kg) of *A. koenigii* was extracted with methanol at room temperature five times, each for five days. The methanol extracts were combined and evaporated under reduced pressure. The residue was suspended in water and extracted with *n*-hexane and dichloromethane, and the organic phases were evaporated under reduced pressure to give *n*-hexane- and dichloromethane-soluble fractions. Half of the *n*-hexane-soluble fraction (12.4 g) was separated by CC over silica gel, eluting with CH_2_Cl_2_-MeOH 29 : 1, 19 : 1, 9 : 1, 6 : 1, 3 : 1, and 1 : 1 to give two fractions (Frs. 1 and 2). Fr. 1 (4.7 g) was separated by CC over silica gel, eluting with *n*-hexane-EtOAc 29 : 1, 19 : 1, 9 : 1, 6 : 1, 3 : 1, and 1 : 1 to give **1** (15.0 mg), **4** (6.0 mg), **5** (8.0 mg), and **8** (5 mg). The water-soluble fraction was passed through a column packed with Diaion HP-20, eluting with 20% and 60% MeOH-H_2_O and MeOH. The fraction eluting with 20% MeOH-H_2_O (0.1 g) was separated by CC over silica gel, eluting with CH_2_Cl_2_-EtOAc 15 : 1, 12 : 1, 9 : 1, 6 : 1, 3 : 1, and 1 : 1 to give **8** (10 mg) and **10** (46 mg). The fraction eluting with 60% MeOH-H_2_O (0.2 g) was separated by CC over silica gel, eluting with CH_2_Cl_2_-MeOH 30 : 1, 25 : 1, 15 : 1, 12 : 1, 9 : 1, 6 : 1, 3 : 1, and 1 : 1 to give **10** (34 mg).

### 2.4. RP-HPLC Analysis of Compounds **1**, **4**, and **5**

Qualitative and quantitative HPLC analysis was performed with an HPLC-DAD Shimadzu SIL-20AC autosampler using a 5 *µ*m particle analytical RP-18 column (4.6 mm × 250 mm). The mobile phase was acetonitrile (HPLC grade)-deionized H_2_O gradient: 56% acetonitrile (0–5 min) and 100% acetonitrile (5.1–15.5 min). The flow rate was 1 mL/min. The injection volume was 20 *μ*L. The column temperature was 30°C. The HPLC conditions were optimized for the baseline, precision, accuracy, repeatability, LOD, and LOQ at the corresponding signal-to-noise ratios 3 and 10 ([Supplementary-material supplementary-material-1]). A wavelength of 306 nm was selected for the detection of methyl flavonols. An accurate weight of the methanol rhizome extract (34.7 mg) was dissolved in 1 mL MeOH of HPLC grade. The sample was passed through a silica gel solid-phase extraction (SPE) cartridge eluting with MeOH to remove impurities. The methanol solution was filtered through a 0.45 *μ*m Millipore membrane filter then analyzed directly by HPLC. The measurements were performed in triplicate to calculate the average values. A calibration curve showing the linear relationship between amounts of the standards injected (*μ*g, *x*-axis) and the peak area (*y*-axis) was constructed. The calibration standards were prepared by serial dilution of 1000 ppm concentration stock solutions of **1**, **4**, and **5** (HPLC purity ≥ 95%) ([Supplementary-material supplementary-material-1]).

### 2.5. X-Ray Crystallographic Analysis of Compounds **1** and **4**

X-ray diffraction data were collected on a Bruker APEX-II CCD diffractometer with Mo K*α* radiation (*λ* = 0.71073 Å). The procedures were done using the following tools: absorption correction: multi-scan, SADABS 2016/2 (Bruker, 2016/2); data collection: APEX3 (Bruker, 2017); cell refinement: SAINT (Bruker, 2001); data reduction: SAINT (Bruker, 2001); programs used to solve and refine structure: SHELXTL (Sheldrick, 2015); molecular graphics: SHELXTL (Sheldrick, 2015); and software used to prepare the material for publication: SHELXTL (Sheldrick, 2015). Reflections were collected at 100 K.

### 2.6. Antimicrobial Activity Assay

The antimicrobial assay was performed in 96-well plates, and minimum inhibitory concentration (MIC) was determined as described by Vanden Berghe and Vlietinck [7]. The bacterial test organisms were cultured on tryptone soya agar (TSB, Sigma-Aldrich) and the test fungi on Sabouraud's dextrose agar (SDB, Sigma-Aldrich). The bacterial inocula were adjusted to yield a density of 1.5 × 10^8^ colony forming units (CFU/mL) (0.5 McFarland standard). The samples were dissolved in dimethylsulfoxide (DMSO) by vortexing and filtered through a 0.02 *μ*m microfilter to produce stock solutions. The wells were filled with 50 *μ*L stock solutions in DMSO and 50 *μ*L organism suspension. The positive controls were prepared in DMSO: ampicillin (50 mM), tetracycline (10 mM), and nystatin (0.04 mM). MIC values of the positive controls were as follows: ampicillin 21.83 *μ*g/mL, tetracycline 6.87 *μ*g/mL, and nystatin 1.44 *μ*g/mL (for yeasts) and 2.89 *μ*g/ml (for fungi). The negative control was DMSO. The wells were allowed to diffuse for 24-hour incubation at 37°C and 48-hour incubation at 30°C for bacteria and fungi, respectively. The results were positive when no growth was observed (CFU < 5). MIC was determined by using serial dilution of the stock solutions. MIC is defined as the lowest concentration of the compounds that showed no growth compared with growth in control (DMSO) wells. All determinations were performed in triplicate.

### 2.7. DPPH Free Radical-Scavenging Assay

Approximately, 3 mL of 0.1 mM solution of 1,1-diphenyl-2-picrylhydrazyl (DPPH) radical in EtOH 96% was each added into six series of prepared concentrations of sample solutions (1 mL in DMSO). The test was performed in triplicate. The solution was mixed vigorously and left to stand at room temperature for 30 min in the dark after which its absorbance was measured spectrophotometrically at 515 nm using an ultraviolet spectrophotometer. DMSO was used as the blank and negative control (1 mL DMSO mixed with 3 mL DPPH), and quercetin was used as the standard. The concentration of the sample required to inhibit 50% of DPPH free radicals is denoted as SC_50_, and the value was determined using TableCurve AISN Software (Jandel Scientific) based on the calculated values of the DPPH scavenging activity (%) of the sample. DPPH scavenging activity (%) was calculated with the following formula: scavenging capacity (SC) (%) = 100 − (*A*1 − *A*0)/*A*_control_ × 100, where *A*0 is the absorbance of the blank (sample in DMSO), *A*_control_ is the absorbance of the negative control, while *A*1 is the absorbance in the presence of the sample [8].

## 3. Results and Discussion

### 3.1. Structure Elucidation of Compounds **1**–**10**

**Figure 1:**
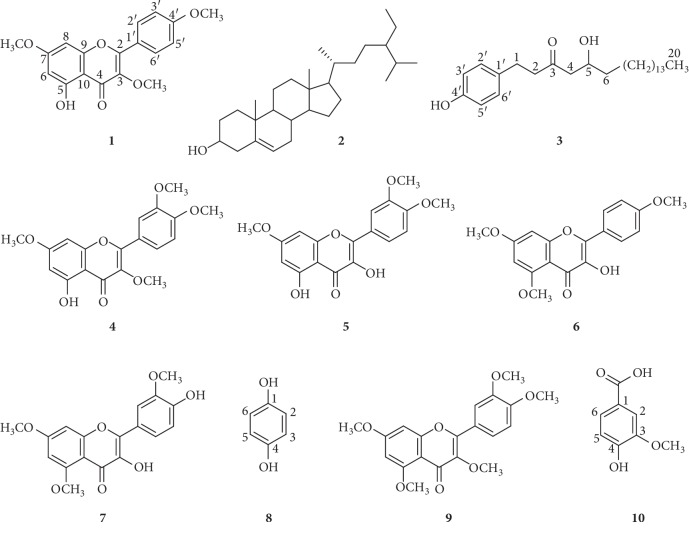
Structures of compounds **1**–**10**.

5-Hydroxy-3,7,4′-trimethoxyflavone (**1**) [5, 9], *β*-sitosterol (**2**) [10], 5-hydroxy-1-(4′-hydroxyphenyl)eicosan-3-one (**3**) [11], retusin (5-hydroxy-3,7,3′,4′-tetramethoxyflavone) (**4**) [5, 12], 3,5-dihydroxy-7,3′,4′-trimethoxyflavone (**5**) [5, 13], 3-hydroxy-5,7,4′-trimethoxyflavone (**6**) [5], 3,4′-dihydroxy-5,7,3′-trimethoxyflavone (**7**), *p*-hydroquinone (**8**) [14], 3,5,7,3′,4′-pentamethoxyflavone (**9**) [5, 15], and vanillic acid (**10**) [16, 17] were isolated from the rhizomes and fruit of *A. koenigii* (Figure 1). The methylated flavonols include kaempferol methyl ethers **1** and **6** and quercetin methyl ethers **4**, **5**, **7**, and **9**. Among the methylated flavonols, compounds **1**, **4**–**7**, and **9** were isolated from the rhizomes and compounds **1**, **4**, and **5** were isolated from the fruit. Compounds **2**, **3**, **7**, **8**, and **10** were reported for the first time from the species. The structures of the compounds were identified using MS and NMR spectroscopy and by comparison of their spectroscopic data with literature values. 2D NOESY and X-ray crystallographic techniques were used to determine the positions of methyl groups in compounds **1**, **4**, **6**, and **7**.

### 3.2. X-Ray Crystallographic Analysis of Methylated Flavonols **1** and **4**

Single crystals of **1** (crystal size 0.43 × 0.23 × 0.12 mm) and **4** (crystal size 0.37 × 0.07 × 0.04 mm) were prepared by recrystallization. We confirmed the locations of the methyl substituents (A-ring 5,7-substitution and B-ring 1,4- and 1,3,4-substitution) in **1** and **4** by X-ray crystallographic analysis. The unit cell of **1** was determined to be the triclinic crystal system, P-1 space group with *Z* = 2 and molecular formula C_18_H_16_O_6_ ([Supplementary-material supplementary-material-1]; [Supplementary-material supplementary-material-1]). The molecule is nonplanar with the dihedral angle between the benzopyranone ring and the 4-substituted phenyl ring of 28.971(6)°. In the crystal, molecules of **1** are linked by weak intermolecular C–H…O hydrogen bonds forming ribbons lying parallel to (100) ([Supplementary-material supplementary-material-1]; [Supplementary-material supplementary-material-1]).

The unit cell of **4** was determined to be orthorhombic crystal system, Pbca space group with *Z* = 47 and molecular formula C_19_H_18_O_7_ ([Supplementary-material supplementary-material-1]; [Supplementary-material supplementary-material-1]). Excluding the methyl moieties of the substituted methoxy groups, the molecule of **4** is essentially planar with non-H atoms exhibiting mean and maximum deviations from coplanarity of 0.0808 and 0.0896 Å, respectively. In the crystal, molecules are linked by weak intermolecular C–H⋯O hydrogen bonds forming ribbons lying parallel to (100) ([Supplementary-material supplementary-material-1]; [Supplementary-material supplementary-material-1]).

### 3.3. HPLC Analysis of Compounds **1**, **4**, and **5**

Previously, compounds **1**, **4**, 3,7-dihydroxy-5,4′-dimethoxyflavone, and 3,7-dihydroxy-5,3′,4′-trimethoxyflavone were analyzed in the seeds and pericarps of *A. koenigii* collected from Guangxi Province and Yunnan Province of China [6]. A typical analytical HPLC chromatogram of the rhizome methanol extract is shown in [Supplementary-material supplementary-material-1]. Compounds **1**, **4**, and 3,5-dihydroxy-7,3′,4′-trimethoxyflavone (**5**) showed three main peaks in the HPLC chromatogram of the rhizome MeOH extract of *A. koenigii* from Vietnam. The retention times of **1**, **4**, and **5** were 12.6, 11.9, and 10.3 min, respectively. According to the HPLC quantification, the flavonoids were present in the rhizomes of *A. koenigii* in dry quantities of 1.81% (**1**), 1.38% (**4**), and 1.76% (**5**). The content of the major compound **4** (retusin) was almost 0.5% in the seeds [6] which is much lower than its content in the rhizomes in the present study.

### 3.4. Antimicrobial Activity of Compounds **1** and **4**–**7**

Five methylated flavonols **1** and **4**–**7** were tested for their antibacterial and antifungal activity as described by Vanden Berghe and Vlietinck [7]. The microorganisms used included *Escherichia coli* (ATCC 25922), *Pseudomonas aeruginosa* (ATCC 25923), *Bacillus subtilis* (ATCC 27212), *Staphylococcus aureus* (ATCC 12222), *Aspergillus niger* (439), *Fusarium oxysporum* (M42), *Candida albicans* (ATCC 7754), and *Saccharomyces cerevisiae* (SH 20). The samples were tested for their antimicrobial activity, and the active samples underwent serial 2-fold dilutions to determine MIC values. The MIC is defined as the lowest concentration of compounds that inhibits any visible growth of microorganisms after incubation. Compounds were tested at the concentrations of 100 *μ*g/mL (**1**, **4**, and **5**) and 50 *μ*g/mL (**6** and **7**); only **4** showed potent activity against *A. niger* with an MIC value of 100 *μ*g/mL (Table 1). Quercetin is a broad-range antibacterial compound; it is active against *S. aureus* (MIC 100 *μ*g/mL), *P. aeruginosa* (MIC 100 *μ*g/mL) [18], and *E. coli* (MIC_50_ 35.76 *μ*g/mL) [19]. In accordance with our results, high levels of methylation in **4**, **5**, and **7** decreased the antibacterial activity, and the presence of the hydroxyl groups at C-3′ and C-4′ in the catechol moiety of the flavonoid B ring was important for the activity, while the free hydroxyl groups at C-3, C-5, and C-4′ were not critical. Kaempferol is much less active than quercetin, showing MIC values against *S. aureus* and *B. cereus* of 500 *μ*g/mL, but it is strongly active against *E. coli* (MIC_50_ 25 *μ*g/mL) [20]. Çitoğlu et al. noted that the presence of free hydroxyl groups at C-5 and C-4′ was not significant for strong antibacterial activity of kaempferol methyl ethers [21]. No activity was observed for **6** showing that the free hydroxyl group at C-3 was not critical for strong antibacterial activity.

### 3.5. Antioxidant Activity of Compounds **4**, **5**, and **7**

The antioxidant activity of three methylated quercetins **4**, **5**, and **7** was evaluated based on the scavenging activity test against DPPH. Compounds **4**, **5**, and **7** were selected because quercetin with its catechol (3,4-dihydroxyphenyl) structure in the flavonoid B ring is a much stronger antioxidant than kaempferol [22]. The samples were dissolved in DMSO, and DPPH was added to ethanol 96%. The absorbance of DPPH was read at *λ* = 515 nm. The tests were triplicated, and the results were averaged (*p* < 0.05). As a result, compounds **4** (30.03%, 100 *μ*g/mL) and **5** (14.20%, 100 *μ*g/mL) showed a lower scavenging activity in comparison with that of quercetin (72.78%, 44 *μ*g/mL) (Table 2). The results provided evidence for the importance of the 3,4-dihydroxyphenyl moiety of the flavonoid B ring in the scavenging activity of quercetin derivatives [22]. No activity was observed for **9** having no free hydroxyl group implying that the C-ring *α*, *β*-unsaturated carbonyl alone is not a requisite for the scavenging activity without the free hydroxyl group at C-3. A slight increase in the scavenging activity of **7** (42.61%, 50 *μ*g/mL) in comparison with those of **4** and **5** was related to the presence of a free hydroxyl group at C-4′ since the 5-hydroxyl group was already blocked and the SC value of **5** with a free hydroxyl group at C-3 was relatively low.

## 4. Conclusions

Two kaempferol methyl ethers: 5-hydroxy-3,7,4′-trimethoxyflavone (**1**) and 3-hydroxy-5,7,4′-trimethoxyflavone (**6**), and four methylated quercetin methyl ethers: retusin (5-hydroxy-3,7,3′,4′-tetramethoxyflavone) (**4**), 3,5-dihydroxy-7,3′,4′-trimethoxyflavone (**5**), 3,4′-dihydroxy-5,7,3′-trimethoxyflavone (**7**), and 3,5,7,3′,4′-pentamethoxyflavone (**9**), were isolated for the first time from the rhizomes of *A. koenigii* from Vietnam. Three methylated compounds **1**, **4**, and **5** were isolated from the fruit. The nonflavonoid compounds *β*-sitosterol (**2**), 5-hydroxy-1-(4′-hydroxyphenyl)eicosan-3-one (**3**), *p*-hydroquinone (**8**), and vanillic acid (**10**) were isolated for the first time from *A. koenigii*. Quantitative HPLC analysis revealed 1.81% of **1**, 1.38% of **4**, and 1.76% of **5** in dry rhizomes. In the antimicrobial activity test, except for the potent activity of **4** against *A. niger* with the MIC value of 100 *μ*g/mL, compounds **1**, **4**, and **5** were not active at 100 *μ*g/mL and **6** and **7** were not active at 50 *μ*g/mL. Weak DPPH scavenging activity of three quercetin methyl ethers **4** (30.03%), **5** (14.20%), and **7** (42.61%) was observed at 100 *μ*g/mL (**4** and **5**) or 50 *μ*g/mL (**7**) concentration.

## Figures and Tables

**Table 1:** Results of the antimicrobial activity test.

No.	Sample	Concentration (*μ*g/mL)	Minimum inhibitory concentration (MIC, *μ*g/mL)							
			*E. coli*	*P. aeruginosa*	*B. subtilis*	*S. aureus*	*A. niger*	*F. oxysporum*	*S. cerevisiae*	*C. albicans*
1	**1**	100	—	—	—	—	—	—	—	—
2	**4**	100	—	—	—	—	100	—	—	—
3	**5**	100	—	—	—	—	—	—	—	—
4	**6**	50	—	—	—	—	—	—	—	—
5	**7**	50	—	—	—	—	—	—	—	—

—: not active.

**Table 2:** Results of the antioxidant activity test.

No.	Sample	Concentration (*μ*g/mL)	Scavenging capacity (SC, %)	SC_50_ (*μ*g/mL)
1	DPPH/EtOH (5 mM ascorbic acid)	15	76.65 ± 0.27	12.67
2	DPPH/EtOH + DMSO	—	0	—
3	Quercetin	44	72.78 ± 0.30	26.86
4	**4**	100	30.03 ± 1.52	—
5	**5**	100	14.20 ± 1.12	—
6	**7**	50	42.61 ± 0.36	—
7	**9**	50	0.00	—

—: not calculated.

## Data Availability

The data used to support the findings of this study are available from the corresponding author upon request.

## Supplementary Materials

Supplementary Materials Figure S1: *Amomum koenigii* J.F.Gmel. Figure S2: the fruit of *Amomum koenigii* J.F.Gmel. Figure S3: standard calibration curves of **1**, **4**, and **5**. Figure S4: molecular conformation of **1** (displacement ellipsoids are drawn at the 50% probability level). Figure S5: a view along the a-axis of the crystal packing of **1**. The hydrogen bonds are shown as dashed lines (see Table S1 for details). Figure S6: molecular conformation of **4** (displacement ellipsoids are drawn at the 50% probability level). Figure S7: a view along the a-axis of the crystal packing of **4**. The hydrogen bonds are shown as dashed lines (see Table 2 for details). Figure S8: HPLC chromatogram of the rhizome methanol extract. Figure S9: ^1^H-NMR of 5-hydroxy-3,7,4′-trimethoxyflavone (**1**). Figure S10: ^1^H-NMR and ^13^C-NMR of 5-hydroxy-1-(4′-hydroxyphenyl)eicosan-3-one (**3**). Figure S11: ^1^H-NMR of 5-hydroxy-3,7,3′,4′-tetramethoxyflavone (**4**). Figure S12: ^1^H-NMR of 3,5-dihydroxy-7,3′,4′-trimethoxyflavone (**5**). Figure S13: ^1^H-NMR and NOESY of 3-hydroxy-5,7,4′-trimethoxyflavone (**6**). Figure S14: ^1^H-NMR and NOESY of 3,4′-dihydroxy-5,7,3′-trimethoxyflavone (**7**). Figure S15: ^1^H-NMR and ^13^C-NMR of *p*-hydroquinone (**8**). Figure S16: ^1^H-NMR and NOESY of 3,5,7,3′,4′-pentamethoxyflavone (**9**). Figure S17: ^1^H-NMR and ^13^C-NMR of vanillic acid (**10**). Table S1: calibration equations, LODs, and LOQs of **1**, **4**, and **5**. Table S2: crystallographic data and structural refinement of **1**. Table S3: hydrogen bond geometry (Å) of **1**. Table S4: crystallographic data and structural refinement of **4**. Table S5: hydrogen bond geometry (Å) of **4**.
