# Supplementary material for: Methylated Flavonols from Amomum koenigii J.F.Gmel. and Their Antimicrobial and Antioxidant Activities
Source: Biochem Res Int. 2020 Feb 18;2020:4812312. doi: 10.1155/2020/4812312 (PMC7049450; doi:10.1155/2020/4812312)
Supplement: Supplementary Materials — Figure S1: Amomum koenigii J.F.Gmel. Figure S2: the fruit of Amomum koenigii J.F.Gmel. Figure S3: standard calibration curves of 1, 4, and 5. Figure S4: molecular conformation of 1 (displacement ellipsoids are drawn at the 50% probability level). Figure S5: a view along the a-axis of the crystal packing of 1. The hydrogen bonds are shown as dashed lines (see Table S1 for details). Figure S6: molecular conformation of 4 (displacement ellipsoids are drawn at the 50% probability level). Figure S7: a view along the a-axis of the crystal packing of 4. The hydrogen bonds are shown as dashed lines (see Table 2 for details). Figure S8: HPLC chromatogram of the rhizome methanol extract. Figure S9: 1H-NMR of 5-hydroxy-3,7,4′-trimethoxyflavone (1). Figure S10: 1H-NMR and 13C-NMR of 5-hydroxy-1-(4′-hydroxyphenyl)eicosan-3-one (3). Figure S11: 1H-NMR of 5-hydroxy-3,7,3′,4′-tetramethoxyflavone (4). Figure S12: 1H-NMR of 3,5-dihydroxy-7,3′,4′-trimethoxyflavone (5). Figure S13: 1H-NMR and NOESY of 3-hydroxy-5,7,4′-trimethoxyflavone (6). Figure S14: 1H-NMR and NOESY of 3,4′-dihydroxy-5,7,3′-trimethoxyflavone (7). Figure S15: 1H-NMR and 13C-NMR of p-hydroquinone (8). Figure S16: 1H-NMR and NOESY of 3,5,7,3′,4′-pentamethoxyflavone (9). Figure S17: 1H-NMR and 13C-NMR of vanillic acid (10). Table S1: calibration equations, LODs, and LOQs of 1, 4, and 5. Table S2: crystallographic data and structural refinement of 1. Table S3: hydrogen bond geometry (Å) of 1. Table S4: crystallographic data and structural refinement of 4. Table S5: hydrogen bond geometry (Å) of 4. [file 4812312.f1.doc]

**SUPPLEMENTARY MATERIALS**

MethyLATED FLAVONOLS FROM *AMOMUM KOENIGII* j.F. GMEL. and THEIR ANTIMICROBIAL and ANTIOXIDANT ACTIVITIES

**Minh Giang Phan,a* Thi Viet Huong Do,a Quoc Binh Nguyenb**

aFaculty of Chemistry, VNU University of Science, Vietnam National University, Hanoi, 19 Le Thanh Tong Street, Hanoi, Vietnam

*bVietnam National Museum of Nature, Vietnam Academy of Science and Technology, 18 Hoang Quoc Viet Street, Hanoi, Vietnam*

**Abstract**

Methylated flavonols form a special group with modulating biological activities in comparison with kaempferol and quercetin. The present study isolated ten compounds including two kaempferol methyl ethers: 5-hydroxy-3,7,4**′**-trimethoxyflavone (**1**), 3-hydroxy-5,7,4′-trimethoxyflavone (**6**); four quercetin methyl ethers: retusin (5-hydroxy-3,7,3**′**,4**′**-tetramethoxyflavone) (**4**), 3,5-dihydroxy-7,3′,4′-trimethoxyflavone (**5**), 3,4′-dihydroxy-5,7,3′-trimethoxyflavone (**7**), 3,5,7,3′,4′-pentamethoxyflavone (**9**); *β*-sitosterol (**2**); 5-hydroxy-1-(4'-hydroxyphenyl)eicosan-3-one (**3**); *p*-hydroquinone (**8**) and vanillic acid (**10**) from the rhizomes and fruit of *Amomum koenigii* J.F. Gmel. (Zingiberaceae). Their structures were determined by MS, NMR and X-ray spectroscopic techniques. Among the methylated flavonols **1**, **4**-**7**, **9** were isolated for the first time from the rhizomes while **1**, **4**, **5** were isolated from the fruit. Compounds **2**, **3**, **7**, **8** and **10** were reported for the first time from the species. Three main methylated flavonols **1**, **4** and **5** were quantitatively analyzed in the rhizomes of *A. koenigii* by RP-HPLC-DAD; their contents were determined to be 1.81% (**1**), 1.38% (**4**) and1.76% (**5**). Antimicrobial assay against *Escherichia coli*, *Pseudomonas aeruginosa*, *Bacillus subtilis*, *Staphylococcus aureus*, *Aspergillus niger*, *Fusarium oxysporum*, *Candida albicans*, and *Saccharomyces cerevisiae* and antioxidant DPPH scavenging test were performed for the isolated methylated flavonols.

*Keywords:* *Amomum koenigii*, Zingiberaceae, methylated flavonols, antibacterial, antifungal, antioxidant

**Figure legend**

Figure S1. *Amomum koenigii* J.F. Gmel.

Figure S2. The fruit of *Amomum koenigii* J.F. Gmel.

Figure S3. Standard calibration curves of **1**, **4** and **5**

Figure S4.Molecular conformation of **1** (Displacement ellipsoids are drawn at the 50% probability level)

Figure S5.A view along the *a* axis of the crystal packing of **1**. The hydrogen bonds are shown as dashed lines (see Table S1 for details)

Figure S6.Molecular conformation of **4** (Displacement ellipsoids are drawn at the 50% probability level)

Figure S7.A view along the a axis of the crystal packing of **4**. The hydrogen bonds are shown as dashed lines (see Table 2 for details)

Figure S8. HPLC chromatogram of the rhizome methanol extract

Figure S9. 1H-NMR of 5-hydroxy-3,7,4′-trimethoxyflavone (**1**)

Figure S10.1H-NMR and 13C-NMR of 5-hydroxy-1-(4'-hydroxyphenyl)eicosan-3-one (**3**)

Figure S11. 1H-NMR of 5-hydroxy-3,7,3′,4′-tetramethoxyflavone (**4**)

Figure S12. 1H-NMR of 3,5-dihydroxy-7,3′,4′-trimethoxyflavone (**5**)

Figure S13. 1H-NMR and NOESY of 3-hydroxy-5,7,4′-trimethoxyflavone (**6**)

Figure S14. 1H-NMR and NOESY of 3,4′-dihydroxy-5,7,3′-trimethoxyflavone (**7**)

Figure S15. 1H-NMR and 13C-NMR of *p*-hydroquinone (**8**)

Figure S16. 1H-NMR and NOESY of 3,5,7,3′,4′-pentamethoxyflavone (**9**)

Figure S17. 1H-NMR and 13C-NMR of vanillic acid (**10**)

**Table legend**

Table S1. Calibration equations, LODs, and LOQs of **1**, **4** and **5**

Table S2.Crystallographic data and structural refinement of **1**

Table S3*.*Hydrogen bonds geometry (Å) of **1**

Table S4.Crystallographic data and structural refinement of **4**

Table S5.Hydrogen bonds geometry (Å) of **4**

*NMR spectroscopic data of compounds* ***1****-****10***

**5-Hydroxy-3,7,4′-trimethoxyflavone (1)**:Yellow amorphour powder.ESI-MS:*m/z* 329.22 [M+H]+, 351.21 [M+Na]+, 327.34 [M−H]−.1H NMR (500 MHz, CDCl3): *δ* (ppm) 12.65 (1H, s, OH-5), 8.07 (2H, dd, *J* = 8.5 Hz, H-2′, H-6′), 7.01 (2H, d, *J* = 8.5 Hz, H-3′, H-5′), 6.43 (1H, d, *J* = 2.0 Hz, H-8), 6.34 (1H, d, *J* = 2.0 Hz, H-6), 3.89 (3H, s, OCH3-7­­), 3.87 (3H, s, OCH3-4′), 3.85 (3H, s, OCH3-3).

***β*-Sitosterol (2)**: White needles. IR (film) γmax (cm–1): 3442, 2956, 2864, 1639, 1458, 1336, 1049, 956.

**5-Hydroxy-1-(4'-hydroxyphenyl)eicosan-3-one (3)**:White amorphous powder.1H NMR (500 MHz, CDCl3): *δ* (ppm) 7.03 (2H, d, *J* = 8.5 Hz, H-2′, H-6′), 6.74 (2H, d, *J* = 8.5 Hz, H-3′, H-5′), 4.01 (1H, m, H-5), 2.83 (2H, t, *J* = 7.5 Hz, CH2-1), 2.72 (2H, t, *J* = 7.5 Hz, CH2-2), 2.57 (1H, dd, *J* = 17.5 Hz, 3.0 Hz, H-4a), 2.48 (1H, dd, *J* = 17.5 Hz, 8.5 Hz, H-4b), 1.48 (1H, m, H-6a), 1.25 (27H, br s, H-6b, from CH2-7 to CH2-19), 0.88 (3H, t, *J* = 6.5 Hz, CH3-20). 13C NMR (125 MHz, CDCl3): *δ* (ppm) 211.4 (C-3), 153.9 (C-4′), 132.9 (C-1′), 129.4 (C-2′, C-6′), 115.4 (C-3′, C-5′), 67.7 (C-5), 49.3 (C-4), 45.3 (C-2), 36.5 (C-6), 31.9 (C-18), 29.7, 29.68, 29.67, 29.6, 29.5, 29.4 (from C-8 to C-17), 28.7 (C-1), 25.5 (C-7), 22.7 (C-19), 14.1 (C-20).

**5-Hydroxy-3,7,3′,4′-tetramethoxyflavone (4)**:Yellow amorphous powder.ESI-MS:*m/z* 359.20 [M+H]+, 381.21 [M+Na]+, 357.29 [M−H]−. 1H NMR (500 MHz, CDCl3): *δ* (ppm) 12.64 (1H, s, OH-5), 7.73 (1H, dd, *J* = 8.5 Hz, 2.0 Hz, H-6′), 7.69 (1H, d, *J* = 2.0 Hz, H-2′), 6.98 (1H, d, *J* = 8.5 Hz, H-5′), 6.45 (1H, d, *J* = 2.0 Hz, H-8), 6.36 (1H, d, *J* = 2.0 Hz, H-6), 3.97 (3H, s, OCH3-3′), 3.96 (3H, s, OCH3-7), 3.88 (3H, s, OCH3-4′), 3.86 (3H, s, OCH3-3).

**3,5-Dihydroxy-7,3′,4′-trimethoxyflavone (5)**: Yellow amorphous powder. ESI-MS:*m/z* 345.22 [M+H]+, 367.17 [M+Na]+, 343.3 [M−H]−.1H NMR (500 MHz, CDCl3): *δ* (ppm) 12.63 (1H, s, OH-5), 7.72 (1H, dd, *J* = 8.5 Hz, 2.0 Hz, H-6′), 7.69 (1H, d, *J* = 2.0 Hz, H-2′), 6.96 (1H, d, *J* = 8.5 Hz, H-5′), 6.45 (1H, d, *J* = 2.0 Hz, H-8), 6.35 (1H, d, *J* = 2.0 Hz, H-6), 5.71 (1H, br s, OH-3), 3.89 (3H, s, OCH3-3′), 3.88 (3H, s, OCH3-7), 3.87 (3H, s, OCH3-4′).

**3-Hydroxy-5,7,4′-trimethoxyflavone (6)**: Yellow amorphous powder. ESI-MS:*m/z* 328.9 [M+H]+. 1H NMR (500 MHz, CDCl3): *δ* (ppm) 8.17 (2H, d, *J* = 9.0 Hz, H-2′, H-6′), 7.03 (2H, d, *J* = 9.0 Hz, H-3′, H-5′), 6.56 (1H, d, *J* = 2.0 Hz, H-8), 6.35 (1H, d, *J* = 2.0 Hz, H-6), 3.98 (3H, s, OCH3-4′), 3.92 (3H, s, OCH3-5), 3.89 (3H, s, OCH3-7).

**3,4′-Dihydroxy-5,7,3′-trimethoxyflavone (7)**: Yellow amorphous powder. ESI-MS:*m/z* 342.9 [M–H]–. 1H NMR (500 MHz, CDCl3): *δ* (ppm) 7.82 (1H, br s, H-2′), 7.80 (1H, dd, *J* = 8.5 Hz, 2.0 Hz, H-6′), 7.00 (1H, d, *J* = 8.5 Hz, H-5′), 6.55 (1H, d, *J* = 2.0 Hz, H-8), 6.36 (1H, d, *J* = 2.0 Hz, H-6), 3.98 (3H, s, OCH3-5), 3.96 (3H, s, OCH3-3′), 3.92 (3H, s, OCH3-7).

***p*-Hydroquinone** **(8)**: White amorphous powder. 1H NMR (500 MHz, CD3OD): *δ* (ppm) 6.64 (4H, s, H-2, H-3, H-5, H-6). 13C NMR (125 MHz, CD3OD): *δ* (ppm) 151.3 (C-1, C-4), 116.8 (C-2, C-3, C-5, C-6).

**3,5,7,3′,4′-Pentamethoxyflavone** (**9**): Yellow amorphous powder. ESI-MS:*m/z* 373.1 [M+H]+. 1H NMR (500 MHz, CDCl3): *δ* (ppm) 7.72 (1H, br s, H-2′), 7.69 (1H, dd, *J* = 8.0 Hz, 2.0 Hz, H-6′), 6.98 (1H, d, *J* = 8.0 Hz, H-5′), 6.50 (1H, d, *J* = 2.0 Hz, H-8), 6.35 (1H, d, *J* = 2.0 Hz, H-6), 3.96 (3×3H, s, OCH3-5, OCH3-4′, OCH3-3′), 3.91 (3H, s, OCH3-7), 3.88 (3H, s, OCH3-3).

**Vanillic acid** (**10**): Yellow amorphous powder. 1H NMR (500 MHz, CDCl3): *δ* (ppm) 7.72 (1H, br d, *J* = 8.5 Hz, H-6), 7.59 (1H, br s, H-2), 6.96 (1H, d, *J* = 8.5 Hz, H-5), 3.97 (3H, s, OCH3-3). 13C NMR (125 MHz, CD3OD): *δ* (ppm) 170.4 (CO2H-1), 152.5 (C-4), 148.6 (C-3), 125.2 (C-6), 123.3 (C-1), 115.8 (C-5), 113.8 (C-2), 56.4 (OCH3-3).


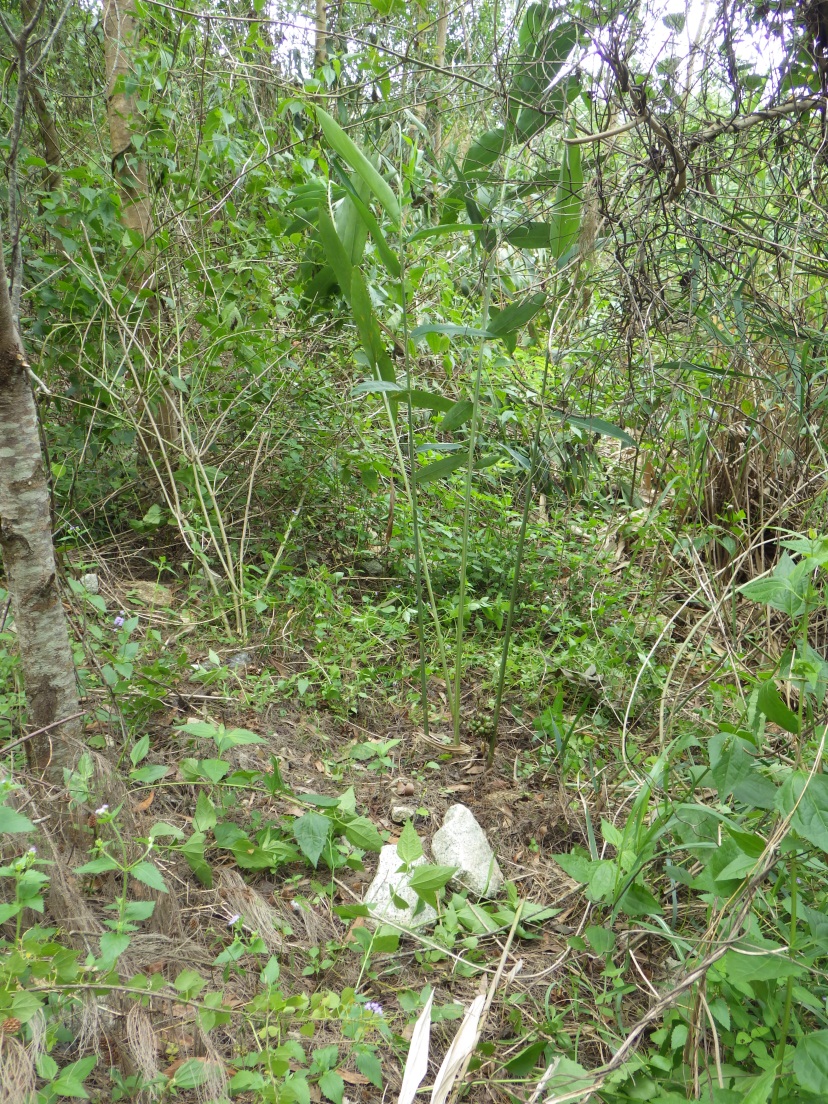


Figure S1. *Amomum koenigii* J.F. Gmel.


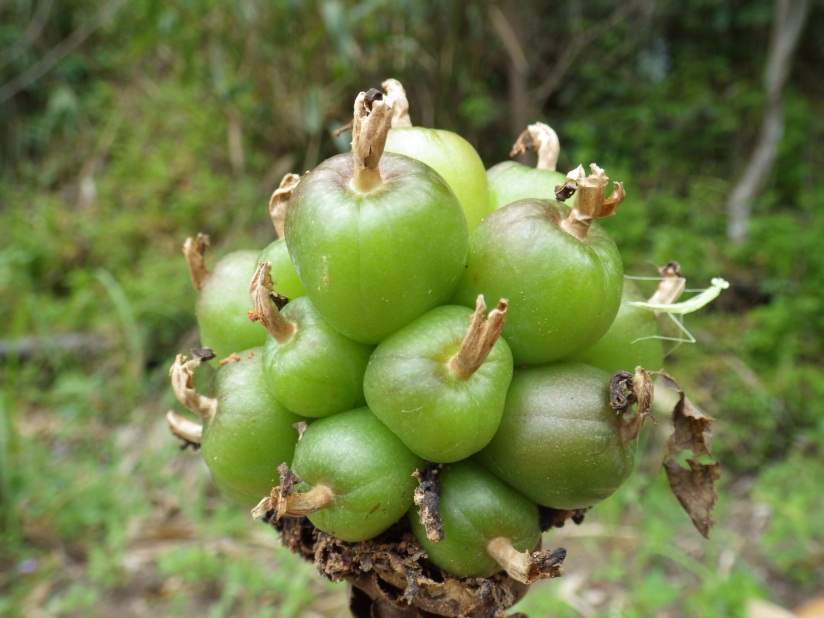


Figure S2. The fruit of *Amomum koenigii* J.F. Gmel.


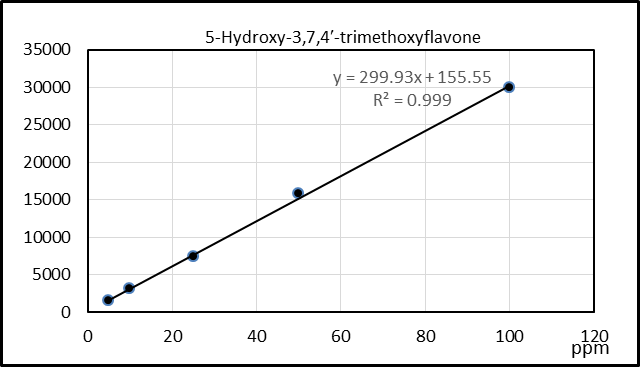


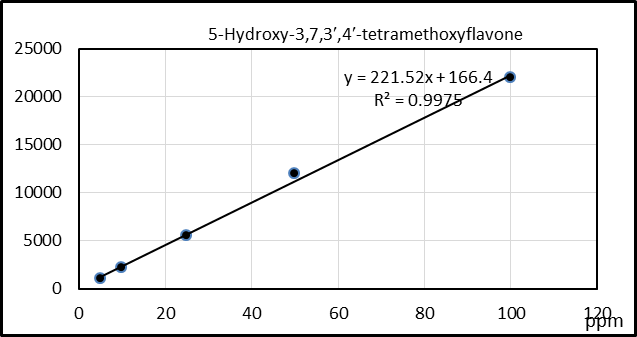


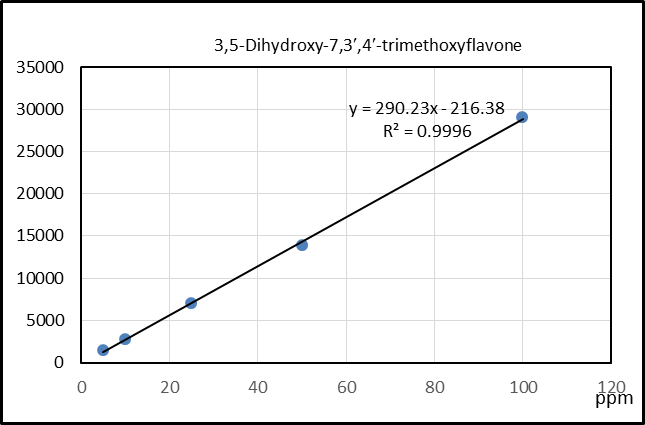


Figure S3. Standard calibration curves of **1**, **4** and **5**


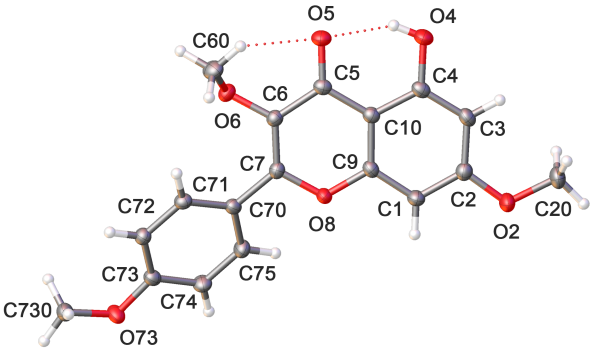


Figure S4.Molecular conformation of **1** (Displacement ellipsoids are drawn at the 50% probability level)


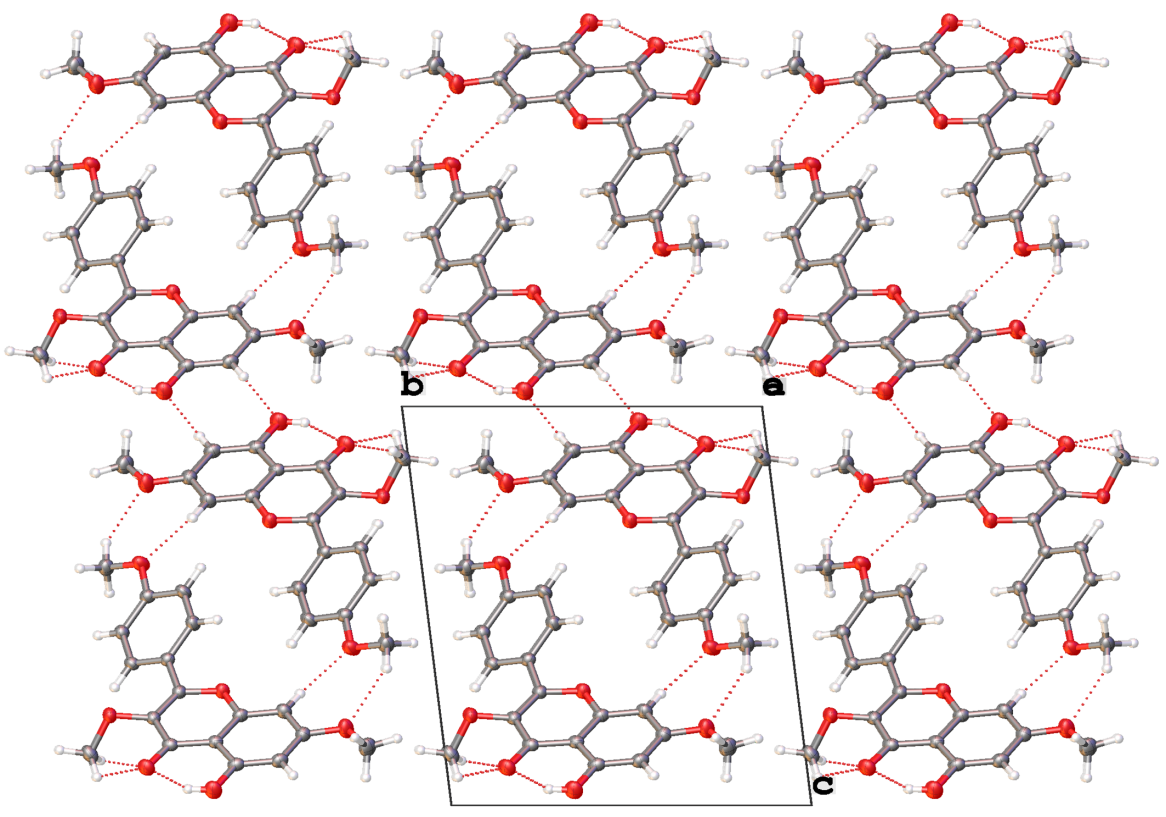


Figure S5.A view along the *a* axis of the crystal packing of **1**. The hydrogen bonds are shown as dashed lines (see Table S1 for details)


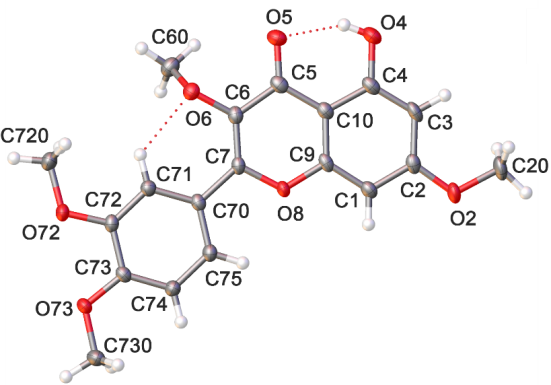


Figure S6.Molecular conformation of **4** (Displacement ellipsoids are drawn at the 50% probability level)


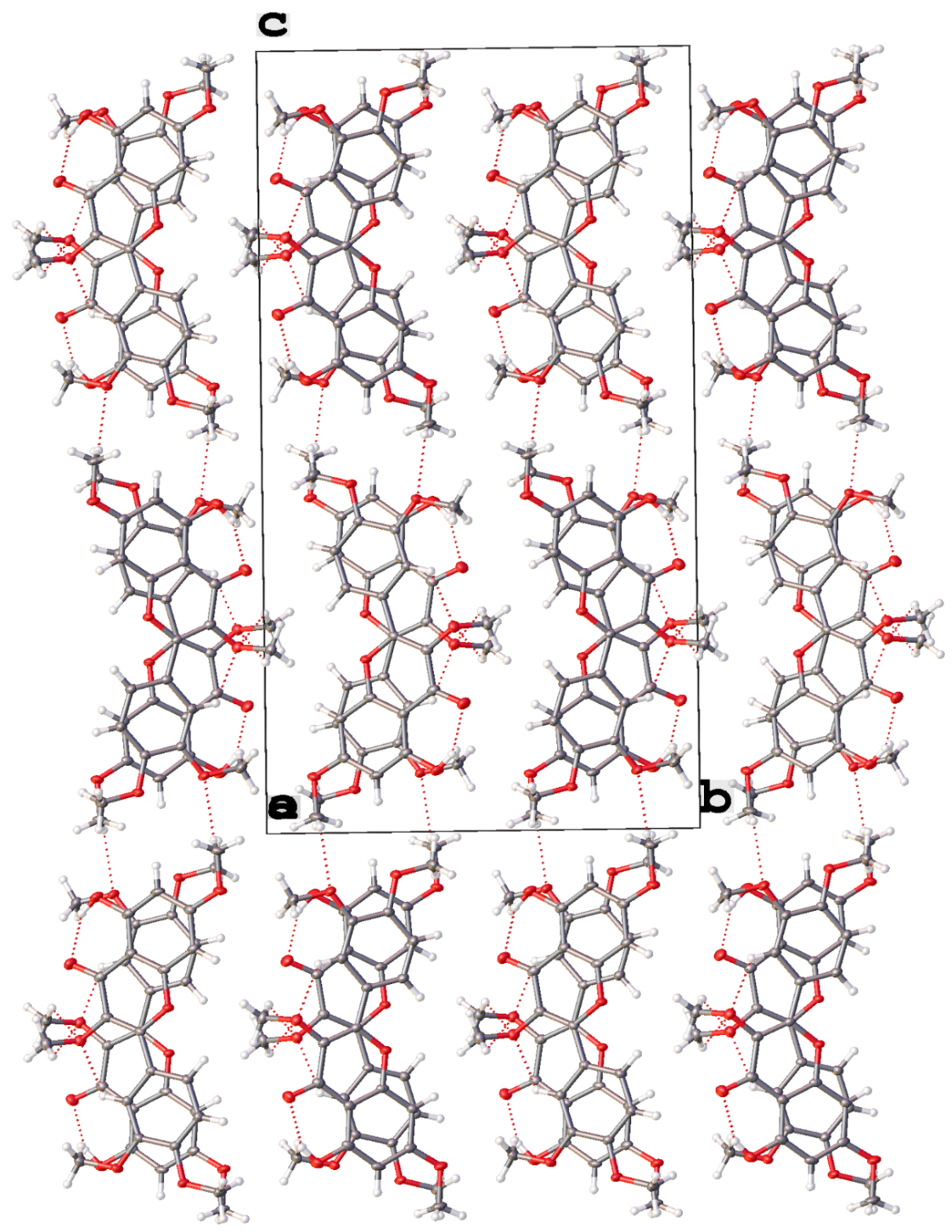


Figure S7.A view along the a axis of the crystal packing of **4**. The hydrogen bonds are shown as dashed lines (see Table 2 for details)


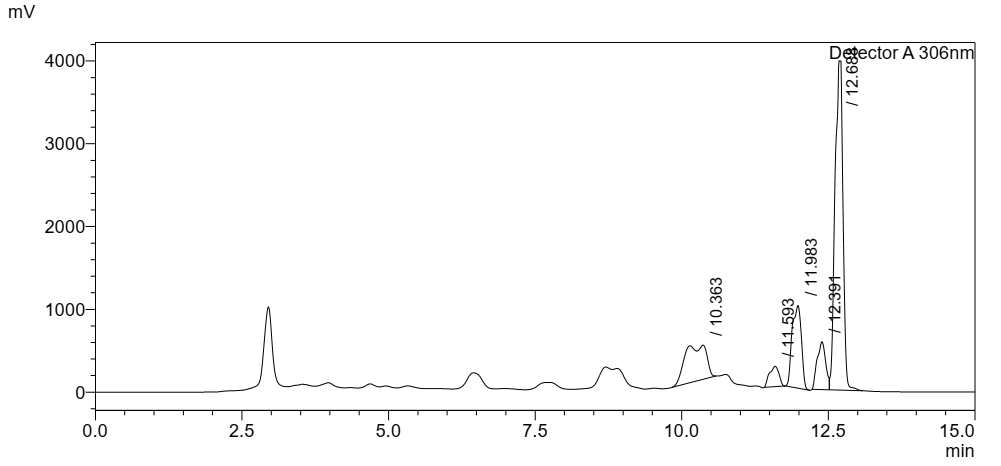


Figure S8. HPLC chromatogram of the rhizome methanol extract


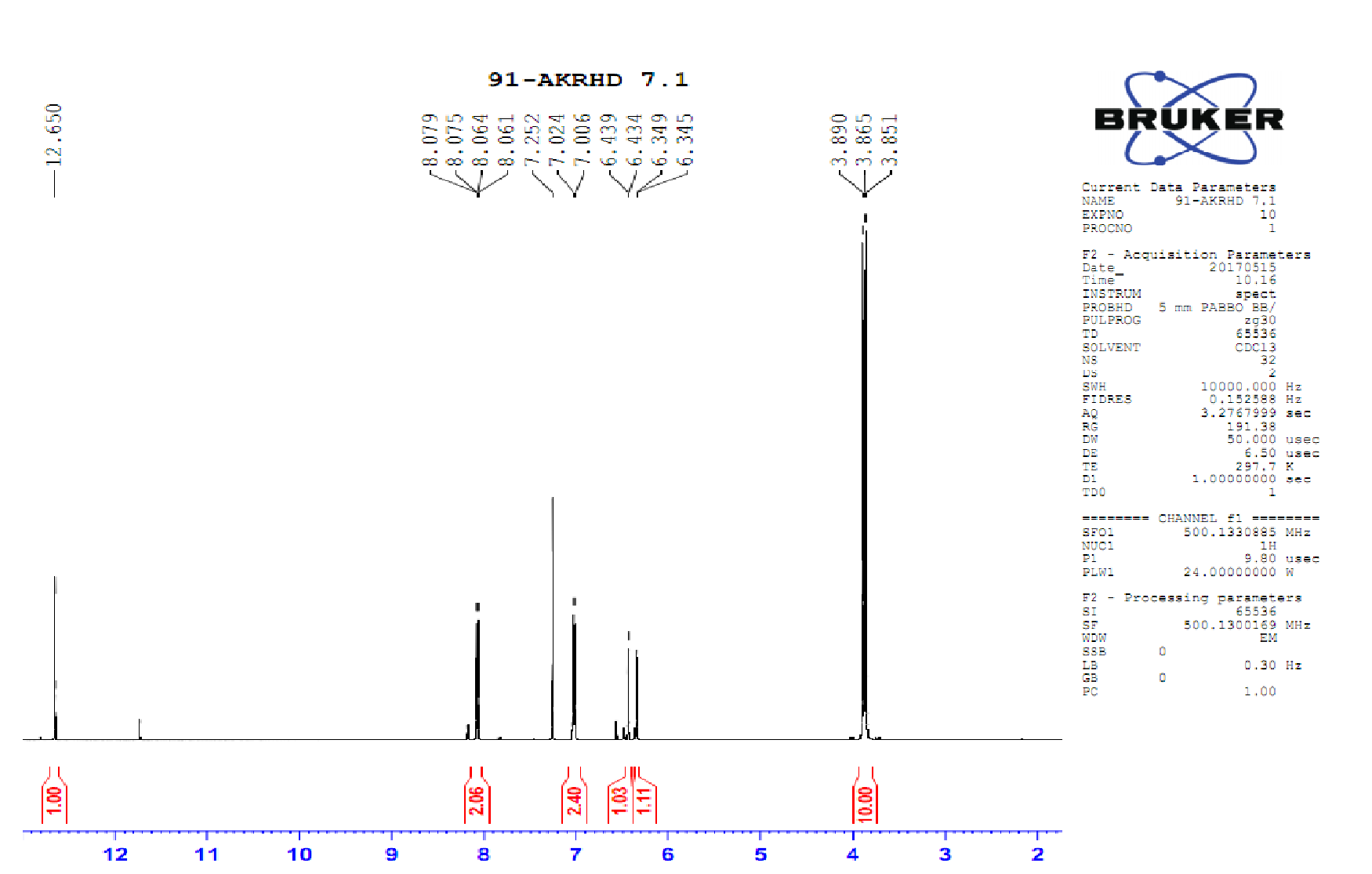


Figure S9. 1H-NMR of 5-hydroxy-3,7,4′-trimethoxyflavone (**1**)

Figure S10.1H-NMR and 13C-NMR of 5-hydroxy-1-(4'-hydroxyphenyl)eicosan-3-one (**3**)


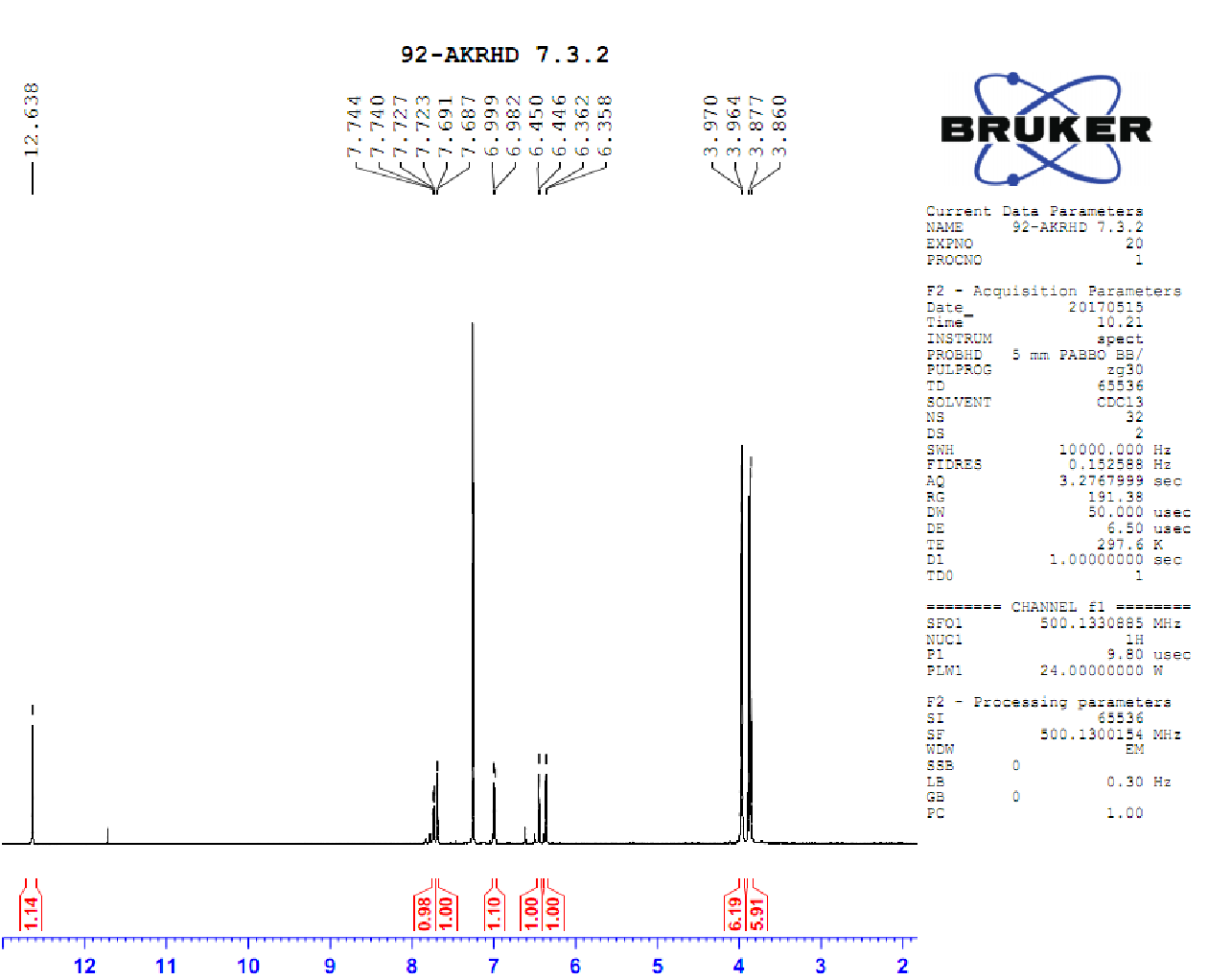


Figure S11. 1H-NMR of 5-hydroxy-3,7,3′,4′-tetramethoxyflavone (**4**)


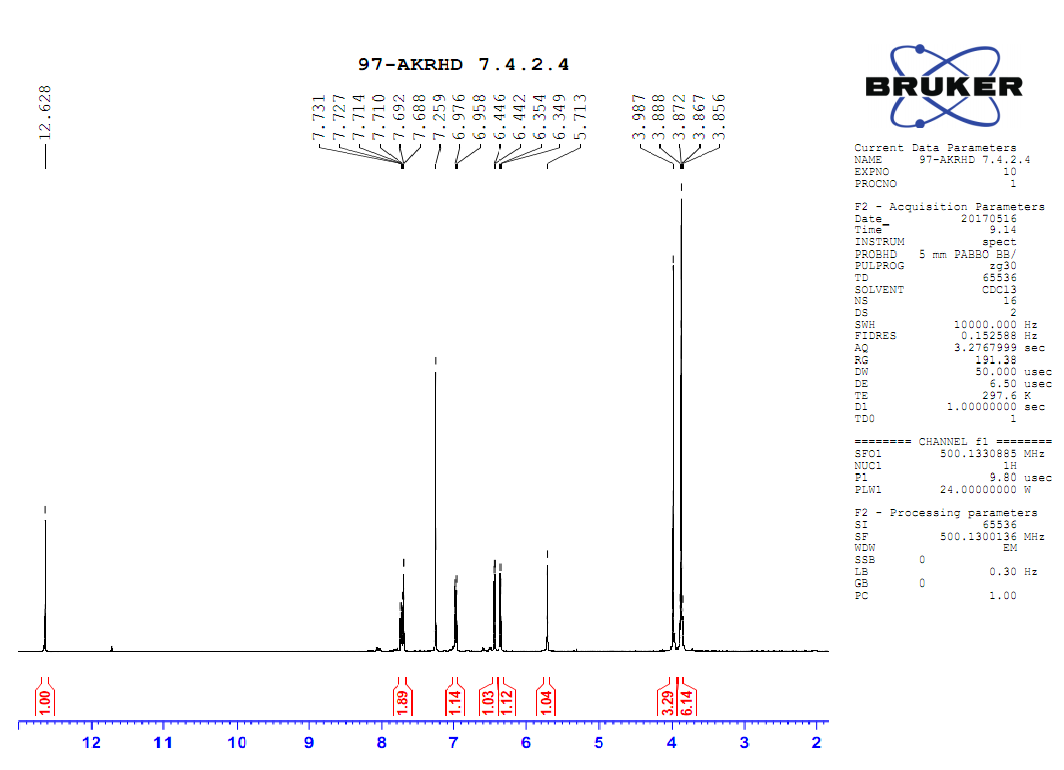


Figure S12. 1H-NMR of 3,5-dihydroxy-7,3′,4′-trimethoxyflavone (**5**)


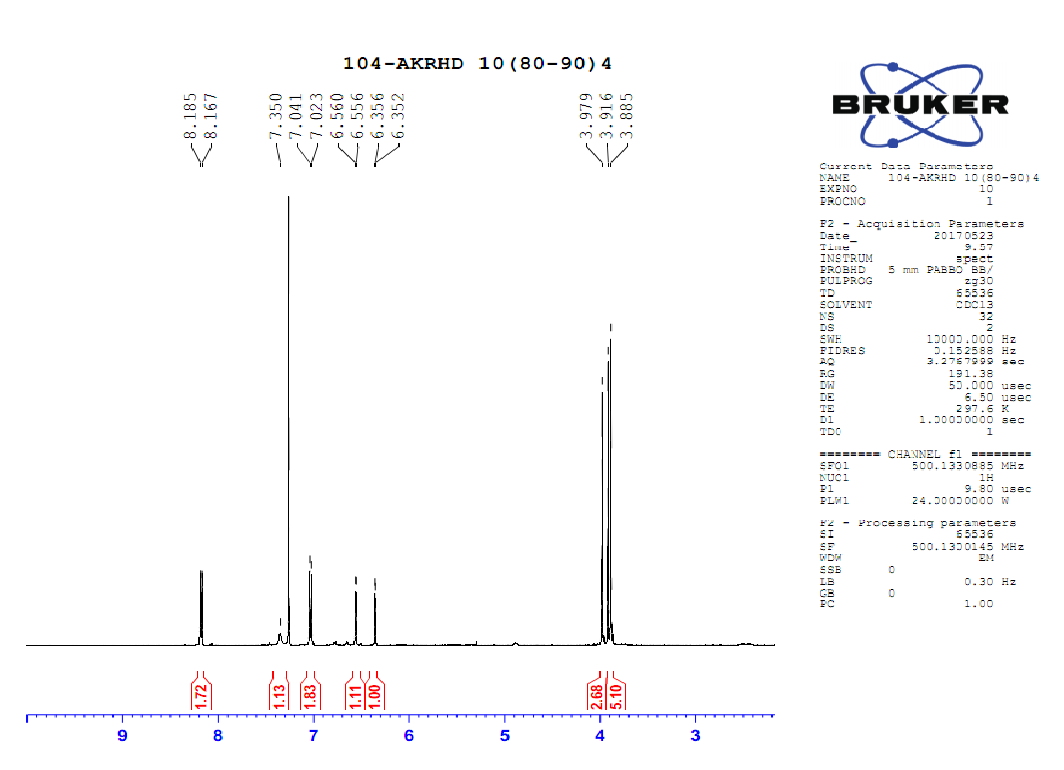


Figure S13. 1H-NMR and NOESY of 3-hydroxy-5,7,4′-trimethoxyflavone (**6**)


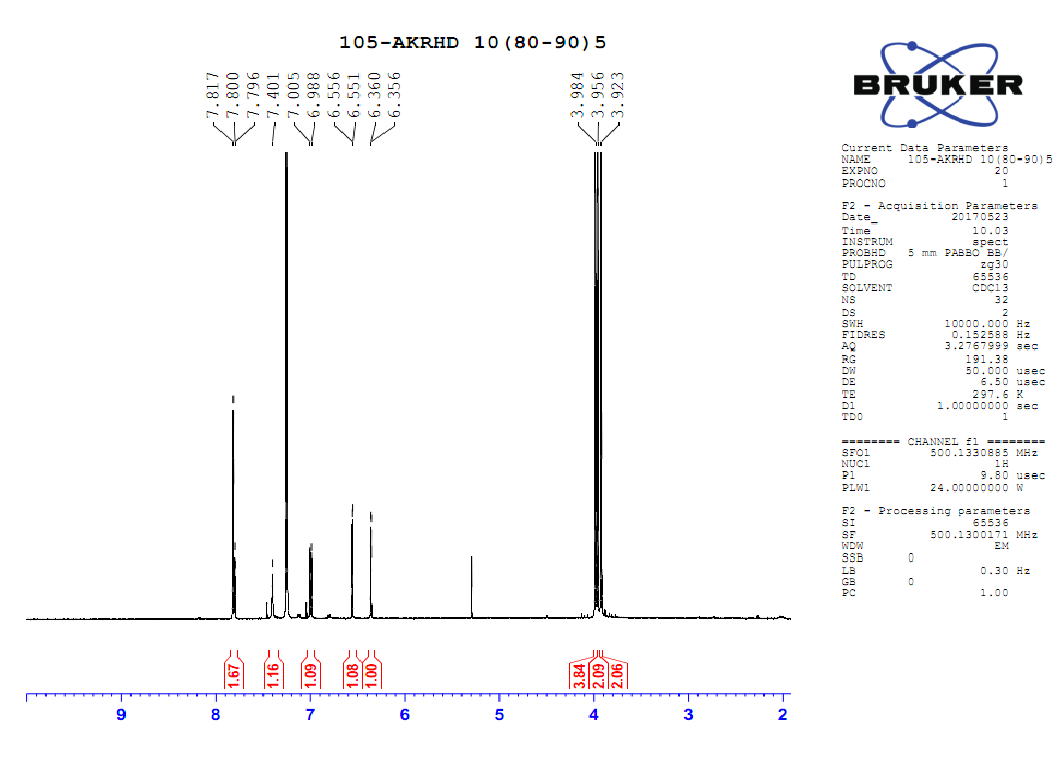


Figure S14. 1H-NMR and NOESY of 3,4′-dihydroxy-5,7,3′-trimethoxyflavone (**7**)

Figure S15. 1H-NMR and 13C-NMR of *p*-hydroquinone (**8**)

Figure S16. 1H-NMR and NOESY of 3,5,7,3′,4′-pentamethoxyflavone (**9**)


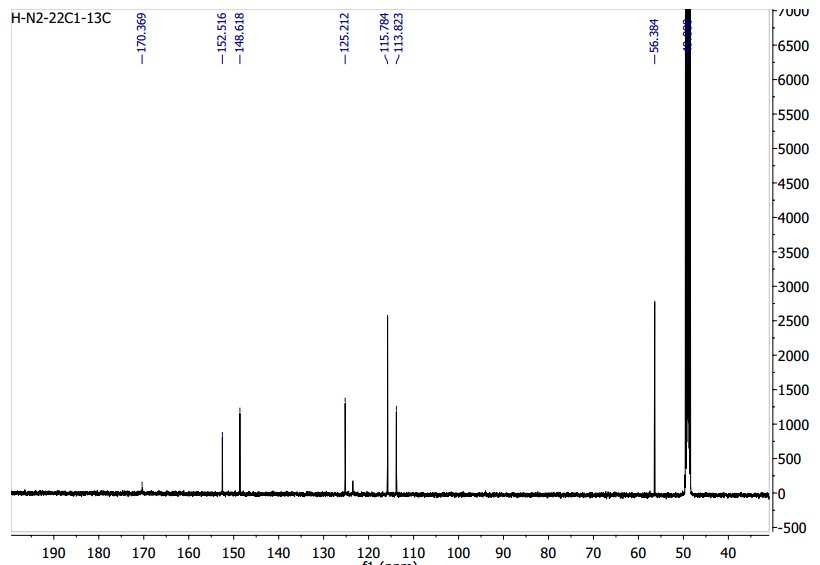


Figure S17. 1H-NMR and 13C-NMR of vanillic acid (**10**)

**Table S1. Calibration equations, LODs, and LOQs of 1, 4 and 5**

| **Compound** | | **Calibration equation** | | **Correlation coefficient** | | **LOD (ppm)** | | **LOQ (ppm)** |
| --- | --- | --- | --- | --- | --- | --- | --- | --- |
|
| **1** | y = 299.93x + 155.55 | | 0.999 | | 0.133 | | 0.443 | |
| **4** | y = 221.52x + 166.4 | | 0.996 | | 0.213 | | 0.7111 | |
| **5** | y = 290.23x – 216.38 | | 0.999 | | 0.089 | | 0.298 | |

Table S2.Crystallographic data and structural refinement of **1**

| Molecular formula | C18H16O6 |
| --- | --- |
| *M*r | 328.31 |
| Crystal system, space group | Triclinic, *P-*1 |
| *a, b, c* (Å) | 5.2724 (8), 11.3197 (16), 12.7616 (18) |
| α, β, γ (°) | 96.194 (4), 99.943 (4), 94.700 (4) |
| *V*(Å3) | 741.87 (19) |
| Z | 2 |
| µ (mm-1) | 0.11 |
| Crystal size (mm) | 0.43 × 0.23 × 0.12 |
| T*min*, T*max* | 0.702, 0.746 |
| No. of measured, independent and observed [*I* > 2σ(*I*)] reflections | 21829, 3747, 2873 |
| *R*int | 0.030 |
| (sin θ/λ)max (Å−1) | 0.673 |
| *R*[*F*2 > 2σ(*F*2)], *wR*(*F*2), *S* | 0.042, 0.110, 1.03 |
| No. of reflections | 3747 |
| No. of parameters | 221 |
| H-atom treatment | H-atom parameters constrained |
| Δρmax, Δρmin (e Å−3) | 0.41, [−](../../../ChienThang/Downloads/tpacfhd4_0m%20_refine_diff_density_min)0.18 |

Table S3*.*Hydrogen bonds geometry (Å, °) of **1**

| D-H…A | d(D-H) | d(H…A) | d(D…A) | <(DHA) |
| --- | --- | --- | --- | --- |
| O(4)-H(4)...O(5) | 0.82 | 1.86 | 2.5953(14) | 148.5 |
| C(60)-H(60B)...O(5) | 0.96 | 2.41 | 3.0180(18) | 121.1 |
| C(1)-H(1)...O(73)i | 0.93 | 2.38 | 3.3016(17) | 168.6 |
| C(730)-H(73A)...O(2)i | 0.96 | 2.57 | 3.4453(18) | 150.9 |
| C(3)-H(3)...O(4)ii | 0.93 | 2.55 | 3.4809(17) | 175.6 |
| C(60)-H(60C)...O(5)iii | 0.96 | 2.45 | 3.3823(19) | 162.8 |

Symmetry codes: (i) −x, −y+1, −z+1; (ii) −x+3, −y+1, −z+2; (iii) x−1, y, z.

Table S4.Crystallographic data and structural refinement of **4**

| Molecular formula | C19H18O7 |
| --- | --- |
| *M*r | 358.33 |
| Crystal system, space group | Orthorhombic, Pbca |
| *a, b, c* (Å) | 7.3869(8), 15.4194(16), 27.855(3) |
| α, β, γ (°) | 90, 90, 90 |
| *V*(Å3) | 3172.7(6) |
| Z | 47 |
| Radiation type | [Mo *K*α](../../../ChienThang/Downloads/tpacfhd4_0m%20_diffrn_radiation_type) |
| µ (mm-1) | 0.16 |
| Crystal size (mm) | 0.37 x 0.07 x 0.04 |
| T*min*, T*max* | 0.6254, 0.7459 |
| No. of measured, independent and observed [[I](../../../ChienThang/Downloads/tpacfhd4_0m%20_reflns_threshold_expression) > 2σ([I](../../../ChienThang/Downloads/tpacfhd4_0m%20_reflns_threshold_expression))] reflections | 34800, 4450, 3289 |
| *R*int | 0.051 |
| (sin θ/λ)max (Å−1) | 0.695 |
| *R*[*F*2 > 2σ(*F*2)], *wR*(*F*2), *S* | 0.049, 0.118, 1.01 |
| No. of reflections | 4450 |
| No. of parameters | 240 |
| H-atom treatment | H-atom parameters constrained |
| Δρmax, Δρmin (e Å−3) | 0.41, [−](../../../ChienThang/Downloads/tpacfhd4_0m%20_refine_diff_density_min)0.21 |

Table S5.Hydrogen bonds geometry (Å, °) of **4**

| D-H…A | d(D-H) | d(H…A) | d(D…A) | <(DHA) |
| --- | --- | --- | --- | --- |
| O(4)-H(4)...O(5) | 0.82 | 1.86 | 2.5956(16) | 148.5 |
| C(71)-H(71)...O(6) | 0.93 | 2.20 | 2.8549(18) | 127.0 |
| C(60)-H(60A)...O(5) | 0.96 | 2.52 | 3.0830(19) | 117.7 |
| C(60)-H(60C)...O(6)#1 | 0.96 | 2.57 | 3.205(2) | 124.0 |
| C(20)-H(20A)...O(72)#2 | 0.96 | 2.47 | 3.204(2) | 133.1 |

Symmetry transformations used to generate equivalent atoms: #1 x+1/2,y,-z+3/2 #2 x,-y+3/2,z-1/2.
